# Prolonged Post-electroconvulsive Therapy Delirium: An Unusual Presentation

**DOI:** 10.7759/cureus.3221

**Published:** 2018-08-28

**Authors:** Ahmad R Khan, Hafsa Mahmood, Saad Wasiq, Hina Saeed

**Affiliations:** 1 Psychiatry, University of North Dakota School of Medicine and Health Sciences, Fargo, USA; 2 Psychiatry, University of Health Sciences, Islamabad, PAK; 3 Psychiatry, Sindh Medical, Ontario, CAN

**Keywords:** ect, delirium, brief pulse, ultrabrief pulse

## Abstract

Electroconvulsive therapy (ECT) is an effective but underutilized modality for the treatment of depression unresponsive to antidepressants. Mild to moderate cognitive impairment is a commonly encountered adverse effect but it normally resolves within hours. We report a case of post-ECT delirium lasting over a course of 14 days with succeeding sessions. Modification of ECT protocol by spacing the intervals of subsequent sessions and switching from bilateral brief pulse to unilateral ultra-brief pulse was found to be effective in reducing the confusion.

## Introduction

Electroconvulsive therapy (ECT) is a recent version of various convulsive therapies that have been used in the treatment of psychiatric illnesses since the 16th century. Under modern medicine, it was first conducted in the year 1938. Initially used for the treatment of schizophrenia, ECT has been demonstrated to be effective in mania, catatonia, severe depression and neurological conditions like Parkinson’s disease and Alzheimer's disease [1,2]. However, its most common use is as a last resort treatment of patients with depression unresponsive to different trials of psychotropic medications. However, despite its established safety and efficacy, it still remains controversial due to misunderstanding in general populace, misinterpretation in media and lack of familiarity of technique among psychiatrists [3]. The exact mechanism by which ECT exhibits antidepressant and anticonvulsant activity is unknown. One theory postulates a decrease in regional brain metabolism [4], the release of hypothalamic hormones or a surge in neurotransmitter availability or receptor sensitivity of amines like dopamine, gamma-aminobutyric acid (GABA), glutamate, and serotonin [5]. Some animal studies have also shown an increase in neurogenesis in the hippocampus [6]. Adverse effects range from mild, i.e., headaches, muscle pain, fatigue and nausea to memory and cognitive impairment [7,8]. Other than these conventional side effects well known to exist with the electroconvulsive therapy, there are certain other complications that may arise secondary to the type of anesthetic used for the procedure. The type of anesthetic used is very important as some of the adverse effects related to anesthetics can make things worse like the use of certain anesthetics has been linked up with prolonged apnea, hyperkalemia and even a condition known as malignant hyperthermia. In the case of this patient with a history of previous malignant hyperthermia, we use non-depolarizing muscle relaxants which have shown a lesser tendency in causing malignant hyperthermia [9]. We are going to discuss a case in which the anticipated retrograde amnesia and delirium extended beyond the average duration.

## Case presentation

We present a case of 68-year-old Caucasian gentleman, a diagnosed case of major depressive disorder, recurrent, severe, without psychotic feature and with anxious distress. He has been suffering from major depressive disorder (MDD) for the last 40 years. He also had post-traumatic stress disorder (PTSD) along with passive suicidal thoughts for a long period of time. Multiple trials of various antidepressants including citalopram, escitalopram, sertraline, paroxetine, and mirtazapine, either used in combinations or as mono-therapy had failed to produce long-term desired effects. The patient was admitted to the psychiatric department on numerous occasions. Psychotherapy was tried but was not effective at all. Medications were discontinued by the patient on account of numerous side effects they produced ranging from a mild headache, nausea, nightmares to confusion. His current spell of depression lasted three months, exhibited by gradual worsening of symptoms, e.g., sleep disturbances, decreased appetite and increased suicidal thoughts. At that time, ECT sessions were planned but never initiated. He is married but states that his social life and family life suffers drastically because of his mood disruptions. He denied any abuse of alcohol or drugs; prescription or recreational. He was losing interest in his current job as well. Concomitantly he suffers from PTSD and anxiety. He also had family history positive for MDD in his mother.

On examination, he was oriented, distressed with prolonged low mood and labile effect. His speech was slow and full of pessimistic thoughts. No cognitive deficits were noted. Based on his history, the team of psychiatrists decided to pursue bilateral brief pulse ECT and discussed it with the patient. Complete medical and neurological investigations were carried out to rule out any comorbidities. Basic metabolic profiles (BMP) including thyroid function tests, electrocardiogram (EKG) and electroencephalogram (EEG) were normal. Informed consent was obtained. Bilateral brief pulse ECT, three times a week (total of 10 sessions), was planned. General anesthesia for a duration of 20-30 minutes was given. Following drugs were administered: Succinylcholine 100 mg, Etomidate 16 mg, Dexamethasone 4 mg and Ondansetron 4 mg for relieving nausea and vomiting post ECT. The first three sessions of ECT were uneventful with no marked post-ECT side effects except for mild headaches. The patient described an alleviation of depressive symptoms and anxiety. However, after the fourth session, the patient experienced delirium (according to diagnostic criteria of DSM-V), starting immediately and lasting for 36 hours. His confusion was characterized by varying degrees of consciousness, disorientation, and difficulty in performing daily activities. Cardiovascular and respiratory parameters like blood pressure, heart rate, and respiratory rate were within normal limits. Metabolic and neurological causes of delirium were ruled out. His delirium improved and he had his fifth ECT session that resulted in profound and prolonged delirium lasting 14 days, relieved without the use of any psychotropic medication. Again, his confusion was characterized by varying degrees of consciousness, disorientation, and difficulty in performing daily activities. A change in ECT protocol, from bilateral brief pulse to right-sided unilateral ultra-brief pulse, was opted for. The first session of unilateral ECT provided significant development. No confusion following procedure was noticed.

With successive session separated by a minimum of three to four days, the patient felt more and more improvement in his depression and anxiety. There was a mild post-ECT confusion after these sessions which improved within a day. He had five sessions of right-sided unilateral ECT. The sixth session was spaced by one week and seventh session by 15 days. He was then put on once a month maintenance ECT. His depression, suicidal ideations, and somatic complaints improved significantly and he was able to live a functional life.

## Discussion

Our case is unique because no obvious cause of prolonged delirium post-ECT could be found. Acute cognitive impairment following ECT is a commonly reported adverse effect especially in the elderly population [10]. Patients with Alzheimer's disease and Parkinson’s disease are more prone to it; possibly due to already present structural/ neurotransmitter changes in their brain [11]. However, physical examination and investigations carried out before and during delirium did not reveal any such factors in our patient. Confusion usually begins immediately after ECT and typically lasts for an hour or two; resolving spontaneously [12]. Acute confusional state lasting less than one hour is a frequently encountered complaint reported in almost 12% post-ECT patients [13, 14].

Management includes close monitoring of the symptoms and no pharmacological intervention is required. In cases of severe delirium agents like nitro, midazolam and antipsychotics can be used. In our patient, there was no impairment after the first few sessions, then a delayed and prolonged confusion was observed after the fourth session and onwards. A similar case of development of delayed de novo postictal confusion after the sixth session has been reported in an elderly patient with any risk factors [15].

Many studies show that the effectiveness of antidepressants can be increased by concomitant use of bilateral or high dose unilateral ECT [16]. However, there are concerns about the augmentation of adverse effects with this approach. One case report of the development of serotonin syndrome by the concomitant use of venlafaxine and ECT has been reported [17]. The American Psychiatric Association Task Force on ECT discourages the concomitant use of antidepressants and ECT [18]. In light of these considerations, no pharmacotherapy was employed in our scenario and ECT solely produced marked beneficial effects.

Among other pharmacological agents, lithium is known to cause prolonged and severe delirium if used along with ECT [19]. One possible explanation of this phenomenon is that ECT results in an increased permeability of the blood-brain barrier hence exaggerated neurotoxic response to lithium.

Typically, more cognitive side effects are seen in bilateral ECT than the unilateral approach [20]. This observation is consistent with our case. Although some mild confusion post ECT was noticed after unilateral therapy, the symptoms were significantly less pronounced and shorter in duration.

Unfortunately, this happens to be one of the most common reasons of foregoing an otherwise safe and effective therapy. Several agents have been studied over the years which can be beneficial during this period. One of them is rivastigmine, an anticholinesterase primarily used in the treatment of Alzheimer’s disease. However, this novel therapy significantly improves the level of functioning in patients with prior structural alterations in the brain. A different type of delirium post ECT has been seen in patients with CVA. There are newer studies in which donepezil has shown beneficial results in the post-ECT delirium patients but still, there are only a few numbers of case reports available on this therapy.

## Conclusions

Based on this case we managed in our setup, we can recommend this therapeutic strategy to all the clinicians who face a similar scenario where patient has prolonged post-ECT delirium phase or agitation. First of all other causes of delirium should be ruled out and if no other cause can be found then we suggest transition from bilateral brief pulse ECT to unilateral ultrabrief pulse ECT along more spacing out of sessions. This may vary from case to case, so further trials are suggested.
